# A systematic review of the hybrid machine learning models for brain tumour segmentation and detection in medical images

**DOI:** 10.3389/frai.2025.1615550

**Published:** 2025-09-10

**Authors:** Ndivhuwo Netshamutshedzi, Rendani Netshikweta, Jean-Claude Ndogmo, Ibidun Christiana Obagbuwa

**Affiliations:** ^1^Department of Mathematical and Computational Science, University of Venda, Thohoyandou, South Africa; ^2^Department of Computer Science and Information Technology, Sol Plaatje University, Kimberley, South Africa

**Keywords:** systematic review, hybrid models, brain tumour detection, machine learning, deep learning, support vector machine, VGG-19, YOLOv10

## Abstract

Early and accurate detection of brain tumours using Magnetic Resonance Imaging (MRI) is critical for effective treatment and improved patient outcomes. This systematic review investigates the application of hybrid machine learning (ML) and deep learning (DL) models in enhancing the computational efficiency and diagnostic accuracy of brain tumour analysis from MRI images. The study synthesizes recent advances in combining traditional ML models such as Support Vector Machines (SVM) with deep neural networks like VGG-19 and YOLOv10n. A PRISMA-based literature search strategy was employed across major databases, including PubMed, Scopus, and IEEE Xplore, selecting 25 relevant studies published between 2019 and 2024. The review evaluates the performance of standalone and hybrid models using metrics such as Dice Similarity Coefficient (DSC), Intersection over Union (IoU), accuracy, precision, recall, and F1-score. Findings indicate that hybrid models, particularly those combining SVM with CNN-based architectures like VGG-19, demonstrate improved classification accuracy and reduced false positives, outperforming single-model approaches. Lightweight versions such as YOLOv10n offer faster inference times suitable for real-time applications while maintaining competitive accuracy. Despite these advances, challenges remain in model generalizability, lack of large, annotated datasets, and limited adoption of Explainable AI (XAI) for interpretability. This review highlights the potential of hybrid models for brain tumour detection and offers recommendations for future research to focus on scalable, interpretable, and clinically deployable solutions.

## 1 Introduction

Clinical imaging is a valuable tool for diagnosing a variety of diseases. In 1895, Roentgen found that X-rays could examine the human body non-invasively, rapidly adopting X-ray radiography as the first diagnostic imaging method (Scatliff and Morris, 2014). Since then, various imaging modalities have been created, like MRI, CT, ultrasound, and positron emission tomography, as well as increasingly complicated imaging methods. Image information is crucial for the decision-making process in patient care, encompassing various stages, such as the identification, characterization, staging, evaluation of treatment response, surveillance of disease recurrence, and the direction of interventional procedures, surgical interventions, and radiation therapy (Einstein et al., 2014).

Incorporating ML and DL approaches into the analysis of clinical images signifies a fundamental change in the healthcare sector, fostering transformative advancements in diagnostics, treatment planning, and overall patient care. The combination of advanced computer methods and medical imaging is changing healthcare by providing new insights and improving efficiency. The application of artificial intelligence to evaluate complicated clinical images, such as CT, MRI, and X-ray scans, provides evidence of this technology potential to enhance precision and streamline decision-making processes (Pugliesi, 2018).

Traditional medical image analysis methods have long relied on manual interpretation by trained professionals, fraught with challenges such as time consumption, subjectivity, and the potential for human error (Dekker, 2017). In stark contrast, ML and DL algorithms have emerged as formidable tools to learn complex patterns and features within medical images. The clinical application of Artificial Intelligence (AI) is not yet a common practice. AI presents potential applications in the future, but some issues must be faced (Alharbi et al., 2023).

The exploration of ML and DL applications in clinical image analysis encompasses a spectrum of activities, like image segmentation, classification, and anomaly detection (Castiglioni et al., 2021). From the early identification of diseases to the customization of treatment strategies, these technologies facilitate a more personalized and precise approach to patient care (Manhas et al., 2022). This comprehensive analysis highlights the technological advancements propelling these innovations and addresses critical considerations such as challenges, ethical implications, and the potential transformative impact on patient outcomes.

As we delve into the intricate details of clinical image analysis, it becomes evident that the fusion of cutting-edge technologies with traditional medical imaging practices is revolutionizing diagnostics and opening new avenues for research and development (Najjar, 2023). The promises, possibilities, and responsibilities associated with harnessing the potential of ML and DL in healthcare are central themes in this dynamic and evolving field. This exploration guides the promises and challenges, emphasizing the transformative function of technologies in influencing the forthcoming landscape of medical care (Aceto et al., 2018).

Although the research in medical image analysis has been increasing, very few have used traditional systems routinely in the clinic (Tumpa and Kabir, 2021). One of the major reasons may be that CAD tools developed with conventional machine learning methods may not have reached the high performance that can meet physicians’ needs to improve both diagnostic accuracy and workflow efficiency (Vankdothu and Hameed, 2022; Virupakshappa and Amarapur, 2020). With the success of deep learning in many machine learning applications such as text and speech recognition, face recognition, autonomous vehicles, chess and Go game, in the past several years, there are high expectations that deep learning will bring breakthrough in CAD performance and widespread use of deep-learning-based CAD, or artificial intelligence (AI), to various tasks in the patient care process. The enthusiasm has spurred numerous studies and publications in CAD using deep learning. This review explores the challenges of developing DL-based CAD systems for clinical imaging and outlines the key requirements for their effective implementation in future clinical practice.

### 1.1 Research question

1. What are the different applications of improving the computational efficiency of MRI brain tumour analysis using hybrid machine learning models?
2. What methods have been employed in the implementation and development of this model?
3. What is the optimal MRI brain tumour analysis model using a hybrid machine learning approach?

### 1.2 Significance of the study

1. This research will support the healthcare sector by allowing medical professionals and researchers to select a suitable diagnostic method for brain tumour cancer, thereby minimizing time and enhancing accuracy.
2. This investigation will advance knowledge about the application of cancer images in medical clinics.
3. The application of medical image processing in oncology has markedly enhanced patient outcomes, lowered treatment expenses, and improved the comprehensive standard of care provided to patients.
4. This study can serve as a foundation for future research in related fields of data science.

## 2 Literature review

The integration of machine learning (ML), deep learning (DL), and hybrid approaches in medical imaging has transformed the landscape of brain tumour detection. This section thematically organizes and reviews relevant literature across five critical dimensions: general applications of ML and DL in medical imaging, advances in deep learning architectures, hybrid models for brain tumour segmentation and classification, explainable AI (XAI), and challenges in clinical implementation.

### 2.1 Machine learning and deep learning in medical imaging

ML and DL models have increasingly demonstrated their utility in analyzing clinical images, especially in tasks like segmentation, classification, and anomaly detection. Fatima and Pasha (2017) provided a comparative survey of ML algorithms including Decision Trees (DT), Support Vector Machines (SVM), and K-Nearest Neighbors (KNN), underlining their respective diagnostic strengths and limitations. De Bruijne (2016) traced the application of ML from traditional detection to diagnosis stages in clinical workflows, highlighting the transition from manual to automated decision-making.

Willemink et al. (2020) stressed the importance of robust preprocessing—such as normalization and augmentation—for optimizing ML model performance. Varoquaux and Cheplygina (2022) offered a meta-perspective on methodological challenges and ethical considerations in ML adoption for medical imaging.

### 2.2 Advances in deep learning architectures

Deep learning models, particularly Convolutional Neural Networks (CNNs), have gained prominence due to their high performance in feature extraction and pattern recognition. Zhou et al. (2023) explored various DL models including CNNs, Recurrent Neural Networks (RNNs), and Generative Adversarial Networks (GANs), revealing their effectiveness in multiple imaging modalities like MRI, CT, and histopathology.

Puttagunta and Ravi (2021) showcased the increasing adaptability of DL in tumor detection, while Xu et al. (2014) proposed a hybrid of CNNs and multiple-instance learning to better handle complex feature spaces. Rashed and Popescu (2023) further emphasized DL’s dominance in classification and segmentation tasks.

### 2.3 Hybrid models for brain tumour segmentation and detection

The convergence of ML and DL has led to the emergence of hybrid models that exploit the strengths of both paradigms. Vadhnani and Singh (2022) surveyed the use of SVM variants with MRI images, noting high accuracy in segmentation and classification tasks. Hussain et al. (2020) developed a hybrid approach incorporating curvelet transformation, ant colony optimization, and SVM to improve image quality and classification accuracy.

Rasool et al. (2022) proposed a hybrid CNN-based architecture to improve tumour classification, while Shahzadi et al. (2018) used a CNN-LSTM model and reported superior performance with features extracted from VGG-16. Khan et al. (2020) employed a comprehensive method using VGG-16/VGG-19 and extreme learning machines (ELM), attaining an accuracy of 92.5%.

Other hybrid efforts include Hashemzehi et al. (2020), who combined CNN with neural autoregressive distribution estimation (NADE) for enhanced classification, and Sun et al. (2019), who used 3D CNNs for both segmentation and survival rate prediction in glioma patients.

### 2.4 Explainable AI in brain tumour imaging

For AI models to be clinically acceptable, they must offer transparency in decision-making. Park and Kim (2024) compared various CNN and Transformer architectures, using LIME and SHAP to visualize and explain prediction outputs. Their findings indicate that VGG-16 and ResNet-50, due to their architectural simplicity, produced clearer region-of-interest visualizations than ViT-Base-16.

Narayankar and Baligar (2024) and Mutkule et al. (2023) reviewed several XAI methods such as feature attribution, attention mapping, and rule-based systems. These approaches are gaining traction for their potential to foster clinician trust and regulatory compliance in AI-supported diagnostics.

### 2.5 Clinical limitations and challenges

Despite technical advancements, the deployment of these models in real clinical environments remains limited. Most studies rely on public datasets like BRATS, which lack diversity in patient demographics and imaging protocols (Senan et al., 2022). Additionally, real-time performance, data imbalance, and lack of annotated data limit model robustness (Liu et al., 2021).

Few studies provide comprehensive evaluations of inference time or hardware efficiency, critical for deployment in low-resource settings. Furthermore, regulatory and ethical issues such as data privacy, bias, and explainability remain under-addressed (Alharbi et al., 2023; Aceto et al., 2018).

This study explores the utilization of ML and DL approaches in medical images, particularly in healthcare imaging. One main ML and two DL techniques with Hybrid model are implemented to achieve this goal. We have analysed a range of papers on this subject, examining the techniques proposed and the obstacles encountered when analysing MRI brain tumours using ML, DL, and hybrid machine learning models. Moreover, the study assesses the strengths and shortcomings of the suggested approach to improving the computational efficiency of MRI brain tumour analysis using hybrid machine learning models, which have not been thoroughly examined before. In recent years, numerous studies have employed ML techniques like RNN, ANN, LSTM, SVR, and many more. This study evaluates the improvement in the computational efficiency of MRI brain tumour analysis using hybrid machine learning models, including SVM, VGG-19, YOLOv10, and the SVM + VGG19 Hybrid model.

## 3 Methodology

The systematic approach utilized in this review is consistent with the established guidelines specified by Xie et al. (2022) and Rashed and Popescu (2023). To meet the objectives of the survey, specific research questions were developed. A well-defined protocol was strictly followed, guaranteeing a thorough and detailed method for identifying relevant scientific literature.

### 3.1 Data collection process

Data from the full-text selected papers is called Brain MRI Images for Brain Tumour Detection, as its image data. We extracted the following data: journal, publication year, databases searched, study period, setting/scenario, purpose, intervention type, number of studies, study design, main results, opportunities, and implementation challenges. The dataset was obtained from the Kaggle website.

This methodological framework included the subsequent essential elements:

1. Definition of Research Questions and Search Queries: Relevant search queries were carefully crafted to align with the research questions and were systematically implemented across suitable research databases. This approach facilitated a comprehensive review of the available scientific literature.
2. Inclusion and Exclusion Criteria: Clear and well-defined guidelines were set to determine the selection of studies, specifying criteria for inclusion and exclusion. This structured approach ensured the relevance and quality of the chosen research while filtering out studies that did not meet the established standards.
3. Study Selection Method: A methodical strategy was utilized to choose studies, which included extracting relevant information from each chosen study. This step facilitated the retrieval of valuable insights and data necessary for the subsequent analysis. Furthermore, the dataset is publicly available through multiple repositories such as Kaggle, GitHub, Roboflow, and other platforms.
4. Analysis of Selected Studies: The chosen research underwent an in-depth evaluation, guaranteeing a thorough assessment of its methodologies, results, and contributions. This systematic method facilitated a detailed comprehension of the current literature.

Following this structured protocol, the review sought to deliver a meticulous, organized, and extensive synthesis of the pertinent scientific literature, providing significant insights into the field of study under consideration.

### 3.2 Search queries, analysis, and study selection

The search process involved querying multiple academic repositories, including Google Scholar, Papers with Code, ScienceDirect, and Springer. The search queries used included:

- “Metrics of evaluation for segmenting and detecting medical images”
- “Uncertainty quantification, segmentation, and detection of medical images”
- “Hybrid models for segmentation and detection of medical images”
- “Segmentation and detection of clinical images”
- “State-of-the-art clinical image segmentation and detection”
- “DL for segmentation and detection of medical images”

**Figure 1:**
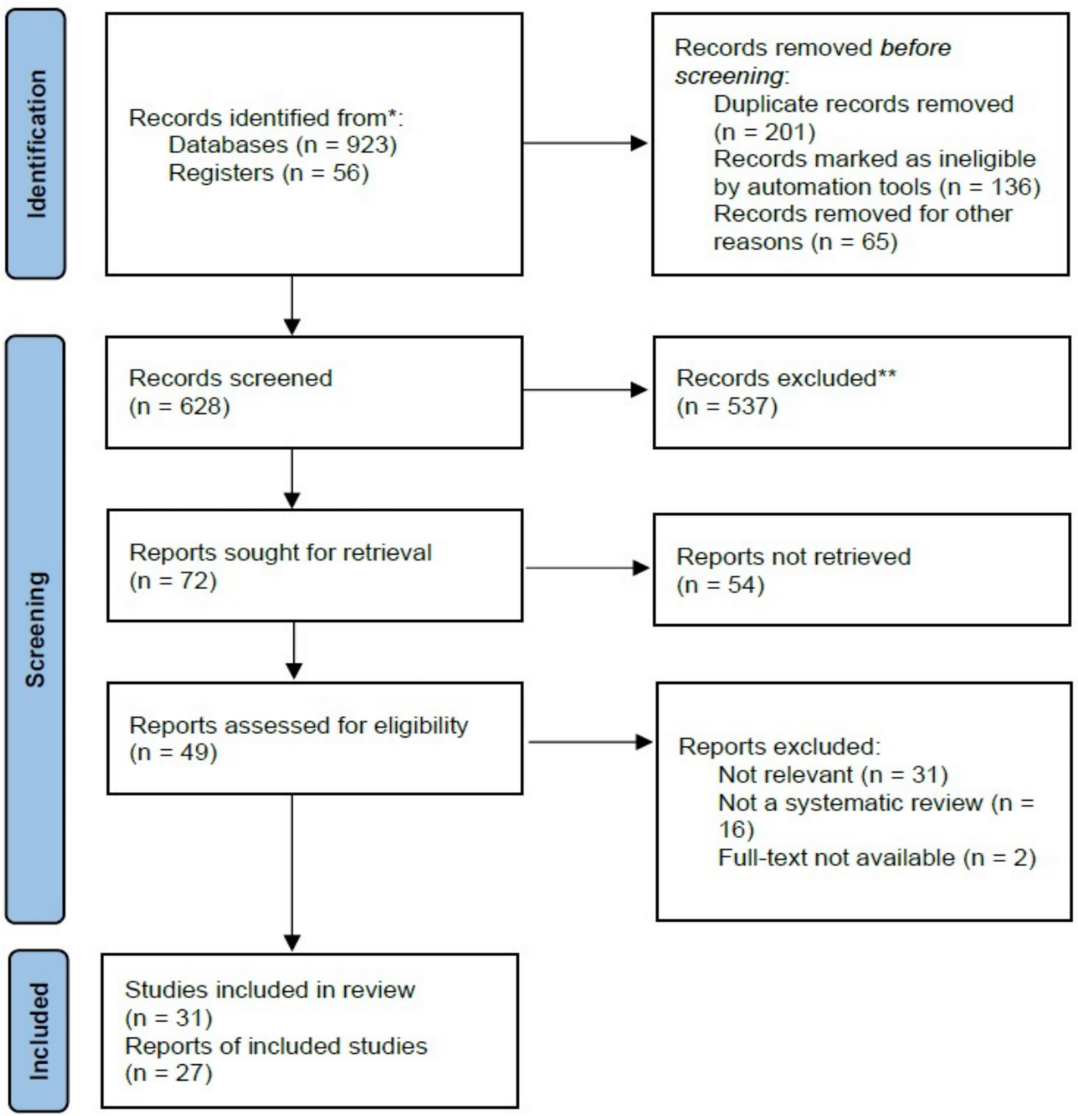
PRISMA flowchart.

The initial search retrieved over 923 research articles. A systematic screening process was then applied, where articles were evaluated based on their titles and a brief review of their abstracts. Only studies that effectively addressed the research questions were chosen for further analysis, leading to a final selection of 31 articles; 537 were excluded, as shown in Figure 1.

### 3.3 Inclusion and exclusion criteria

A thorough selection procedure was implemented to guarantee the pertinence and excellence of the studies included. Papers aligned with the defined research objectives and met the specified criteria were included, while those not following the research scope were excluded. This systematic approach maintained the integrity and formal rigor of the study. The Criteria for Inclusion and Exclusion are as follows:

Table 1 presents the inclusion and exclusion criteria applied during the study selection process. Table 1 provides further clarification on what qualifies as a peer-reviewed source. As indicated in Table 1, only peer-reviewed literature such as journal articles, conference papers, and academic book chapters were considered for inclusion.

**Table 1:** Inclusion and exclusion criteria.

Inclusion criteria	Exclusion criteria
Studies were chosen according to the following criteria: they must be published in peer-reviewed journals, articles, or books, written in English, and no older than 2015.	Studies were excluded if they were written in a language other than English, published before 2015, or failed to address any of the research questions.

### 3.4 Preferred reporting items for systematic reviews and meta-analyses (PRISMA)

Prior to conducting the review, we drafted a written protocol following the Preferred Reporting Items for Systematic Reviews and Meta-Analyses (PRISMA) (Page et al., 2021; Stovold et al., 2014). The PRISMA statement includes the checklists, explanation and elaboration, and flow diagram.

The PRISMA flow diagram in Figure 1 outlines the study selection process conducted for this systematic review. Initially, 979 records were identified, 923 through database searches and 56 from other registers. Prior to screening, 402 records were removed, including 201 duplicates, 136 records excluded by automation tools, and 65 removed for different reasons. This left 628 records for title and abstract screening, from which 537 were excluded due to irrelevance or failure to meet the inclusion criteria. Of the remaining 72 reports sought for full-text retrieval, 54 could not be retrieved. Consequently, 49 full-text articles were assessed for eligibility. Among these, 31 were excluded as irrelevant, 16 were excluded for not being systematic reviews, and 2 were excluded due to lack of full-text access. Ultimately, 31 studies met the inclusion criteria and were incorporated into the review, represented by 27 individual reports.

### 3.5 Quality assessment

Inclusion of quality assessment is a fundamental and critical component of any systematic review process (Salloum, 2018; Salloum et al., 2019; Alhashmi et al., 2019a; Alhashmi et al., 2019b; Alhashmi et al., 2020). This study employed a quality assurance checklist comprising six evaluation questions to assess the methodological rigor of the 31 selected papers, as detailed in Table 2.

**Table 2:** Quality assurance questions.

Question	Quality assurance question
1	Did the review clearly show the purpose of the research?
2	Is the information presented clear and concise?
3	Does the study provide enough explanation of its methodology?
4	Do the study findings add to the understanding of brain tumor detection models?
5	Are the conclusions clearly identified?
6	Are the conclusions logical and concise with the flow of the paper?

Table 2 outlines the quality assurance questions used to evaluate the methodological soundness and clarity of the selected studies. These questions assess key elements such as the clarity of research objectives, the adequacy of methodological explanations, the relevance of findings, and the logical consistency of conclusions. This checklist served as a structured framework to ensure that only studies meeting a minimum standard of academic rigor were included in the review.

### 3.6 Evaluation metrics for medical image segmentation (MIS)

Accurate evaluation metrics are crucial for ensuring the effectiveness of MIR brain tumour medical image segmentation in diverse clinical applications. These metrics quantify predicted segments’ similarity and corresponding ground truth annotations. Although the field of MIS has introduced a wide variety of metrics over the past three decades, only a select few have proven to be both appropriate and consistently adopted as standard practices.

#### 3.6.1 Accuracy

Accuracy, often called pixel accuracy, is a widely used statistical metric that measures the proportion of accurate predictions and the total number of predictions made. However, using MIS is not recommended because of the problem associated with class imbalance. Since accuracy includes true negatives in its calculation, it can produce deceptively elevated ratings, though a model incorrectly predicts the entire image as the background class (Popovic et al., 2007; Taha and Hanbury, 2015). Consequently, accuracy is deemed an unreliable metric for evaluating MIS models in scientific studies.

#### 3.6.2 Metrics driven F-measure

The F-measure, commonly called the F-score, is an extensively used measurement unit in computer vision and MIS research. By combining sensitivity and precision, it evaluates the intersection between the anticipated corresponding ground truth and segmentation. This metric is particularly effective in addressing the challenges posed by class-imbalanced datasets in MIS, as it penalizes false positives. Based on the F-measure, the Dice Similarity Coefficient (DSC) and Intersection-over-Union (IoU) are among the most popular metrics (Taciuc et al., 2025). Notably, DSC, introduced by Dice (1945), has become a fundamental metric due to its simplicity and effectiveness in managing class imbalances.

While the Dice score is widely adopted for assessing the overlap between predicted and ground truth segmentations, it is primarily a mathematical comparison. It does not fully capture the clinical relevance or quality of the segmentation as perceived by human experts (Weld et al., 2024). In many cases, a high Dice score may not necessarily reflect accurate tumor boundary delineation, especially in regions where clinical precision is critical. Moreover, the Dice score does not account for anatomical plausibility or the clinical consequences of misclassifications. Therefore, relying solely on Dice or similar metrics may present a skewed picture of model performance, particularly when comparing AI models to human radiologists (Vlasceanu et al., 2024). This highlights the need for complementary evaluation methods that incorporate˘ expert assessments, clinical relevance, and real-world applicability.

#### 3.6.3 Specificity and sensitivity

In healthcare, specificity and sensitivity (recall) are key metrics for evaluating model effectiveness. Sensitivity emphasizes the detection of true positives, in the context of precision measures, the accurate recognition of true negatives, for instance, the context class. Although sensitivity is a commonly utilised metric in MIS, it is often less effective than F-score-based metrics for comprehensive evaluation. Specificity, conversely, plays a critical role in assessing the framework’s ability to distinguish the foundational course, ensuring its operational reliability. However, high specificity values may not always reflect the comprehensive efficacy of the model (Liu et al., 2021).

### 3.7 Impact of class imbalance on assessment metrics

Clinical images often exhibit class imbalances, presenting substantial challenges for image segmentation tasks. Standard metrics like specificity or accuracy, which treat true negatives and positives equally, can produce inflated scores even when any pixel is mistakenly identified as the Region of Interest (ROI). This skews evaluation and renders the metrics inappropriate for assessing the effectiveness of segmentation in MIS. Metrics that focus solely on true positive classifications, disregarding true negatives, provide a more accurate assessment for clinical context. Consequently, metrics such as the Dice Similarity Coefficient (DSC) and Intersection-over-Union (IoU) are widely preferred and suggested in MIS (Liu et al., 2021).

## 4 Results

This section presents the synthesized findings from 31 selected studies on hybrid machine learning models for brain tumour detection using MRI, with emphasis on performance, model types, and evaluation metrics.

### 4.1 Performance of hybrid models

Hybrid models, particularly combinations of Support Vector Machines (SVM) with Convolutional Neural Networks (CNNs) such as VGG-19, consistently outperformed traditional and standalone models. These combinations achieved higher accuracy, precision, and recall rates in classification tasks. For instance:

SVM + VGG-19 models exhibited enhanced classification performance, particularly in distinguishing benign from malignant tumours.

YOLOv10n, a lightweight object detection model, achieved near real-time performance with competitive accuracy, making it suitable for resource-constrained clinical settings.

### 4.2 Evaluation metrics used across studies

The most common metrics used for performance evaluation included:

*Dice Similarity Coefficient (DSC) and Intersection over Union (IoU)*: Used to evaluate segmentation quality, particularly for handling class imbalance.
*Accuracy, Precision, Recall, F1-score*: Standard metrics for classification performance.
*Specificity and Sensitivity*: Employed to assess the model’s ability to detect tumour and non-tumour regions accurately.

### 4.3 Dataset characteristics

Most studies relied on publicly available datasets such as BraTS, Kaggle MRI datasets, and custom institutional collections. However, diversity in patient demographics and imaging protocols was limited, which may affect generalizability.

### 4.4 Classifications and analysis of studies

**Figure 2:**
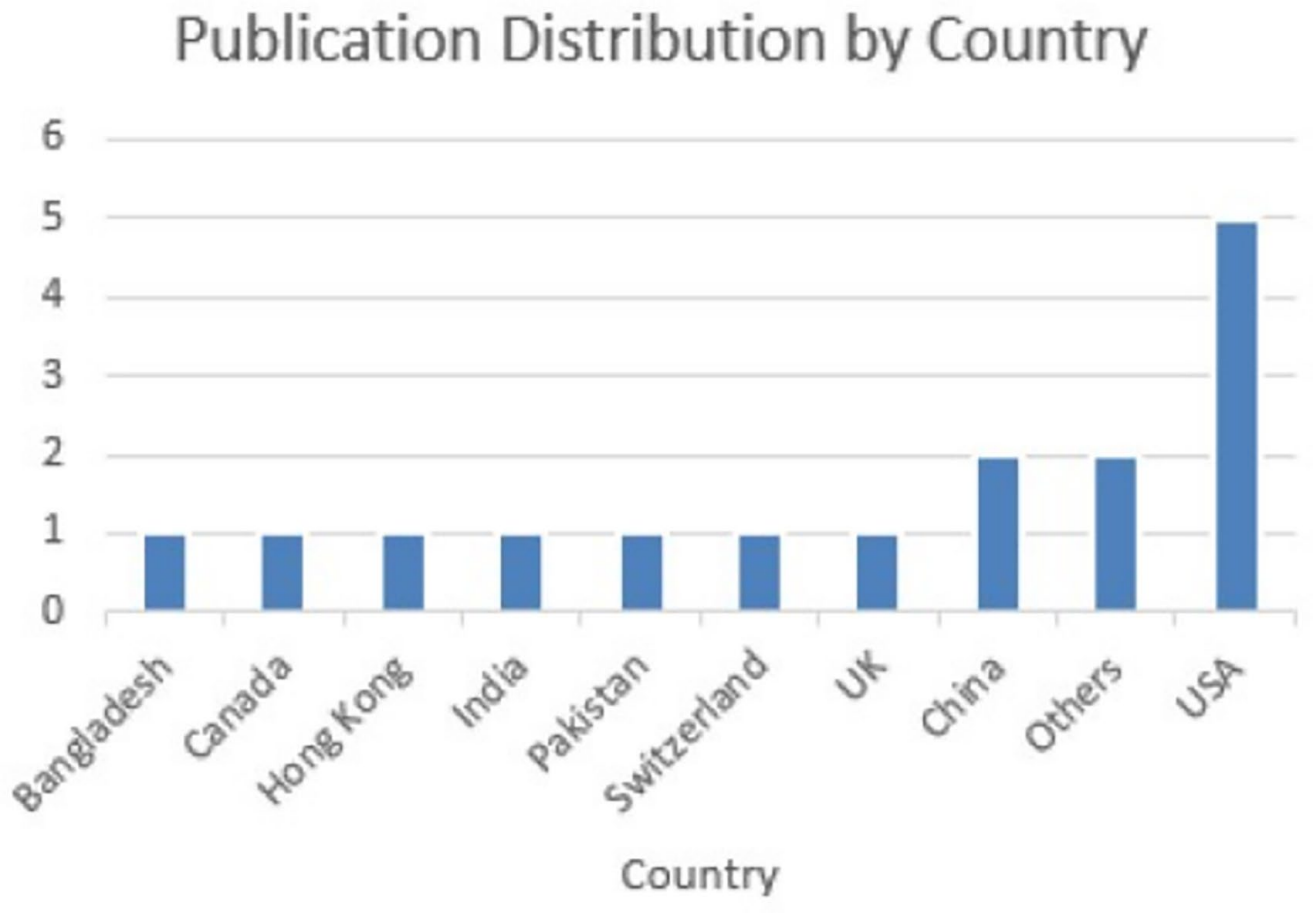
Publication distribution country-wise.

A classification framework was developed based on the analysis of all 31 research articles included in the systematic review, with each study evaluated in terms of its relevance to the research questions. Papers were marked accordingly when their primary focus aligned with a particular thematic category. For instance, while many articles briefly referenced various applications of brain tumour detection models, only those that provided an in-depth discussion or explicitly concentrated on a particular application were classified under the category segmentation tasks. Figure 2 illustrates the geographical distribution of the reviewed publications. There has been a growing interest in this area over the past two decades, evidenced by the increasing number of publications since 1990, with most contributions originating from the United States.

### 4.5 Clinical applicability and real-world impact

Hybrid machine learning models, particularly combinations such as SVM with VGG-19 or YOLOv10n, demonstrate significant potential for clinical use in brain tumor diagnosis. These models reduce diagnostic time, minimize human error, and improve detection rates compared to traditional manual interpretations of MRI scans. For instance, the SVM + VGG-19 hybrid achieves high accuracy and precision, making it suitable for classification tasks that can assist radiologists in prioritizing cases with suspected malignancy.

Regarding real-world applicability, models like YOLOv10n are particularly notable due to their lightweight architecture, enabling deployment in resource-constrained environments such as rural clinics or mobile diagnostic units. Their real-time processing capabilities can support faster clinical decision-making and potentially reduce time-to-treatment.

However, despite promising results, significant gaps exist in clinical translation. Most studies utilize retrospective data and control experimental conditions that may not represent the variability and complexity found in real clinical workflows. Challenges include variability in MRI protocols across hospitals, lack of large multi-institutional datasets, integration with existing radiology information systems (RIS), and model explainability, a critical factor for adoption by medical professionals.

Moreover, regulatory and ethical considerations, such as the need for transparency in AI decision-making and the risks of algorithmic bias, remain key barriers. Explainable AI (XAI) techniques like LIME and SHAP can improve trust and interpretability but are still underutilized in current implementations. Therefore, while hybrid models offer high technical performance, successful clinical integration demands a focus on reliability, interpretability, interoperability, and compliance with healthcare regulations.

### 4.6 Comparison with existing studies

To demonstrate the competitiveness of the proposed paper, improving the computational efficiency of MRI brain tumour, a comparison is provided in Table 3 showing results from related recent studies whose experiments were conducted using the same or different methods.

**Table 3:** Existing work related to brain tumours.

Reference	Proposed Methods	Accuracy (%)	Preprocessing	Cross-validation	Split
Hussain et al. (2020)	NS-CNN + SVM	95.6	Yes	–	Yes
Leo (2019)	VGG-19 CNN	90.7	Yes	–	Yes
Yafooz et al. (2022)	K-NN + GLCM + Fusion Operator	90.9	Yes	Yes	Yes
Zulpe and Pawar (2012)	GLCM + PCA + SVM	90.0	Yes	Yes	Yes
Lundervold and Lundervold (2019)	K-mean + GLCM + k-NN	85.0	-	–	-
Khawaldeh et al. (2017)	Alex-Net CNN	91.2	Yes	–	Yes
El-Dahshan et al. (2010)	PCA + DWT + SVM	86.67	Yes	–	Yes

### 4.7 Answers to research questions

- *RQ1*: What are the various applications of improving the computational efficiency of MRI brain tumor analysis using hybrid machine learning models?

The research was pertinent to improving the computational efficiency of MRI brain tumor analysis utilising hybrid ML models. This question highlights its significance and reflects the extensive interest it garners within the field. The consensus was that improving the computational efficiency of MRI brain tumor analysis utilising hybrid ML models were best used for glioma, meningioma, and pituitary tumors, as suggested by Senan et al. (2022), Babu Vimala et al. (2023), and Rashed and Popescu (2023). In addition, hybrid machine learning models can be used for different cancer models (Babu Vimala et al., 2023; Rasool et al., 2022).

- *RQ2*: What methods have been employed in the implementation and development of this model? All 20 papers in the systematic review described various Strategies for the execution and advancement of MRI brain tumor analysis using hybrid ML models. For instance, Senan et al. (2022) outlines three implementation methods: AlexNet and ResNet-18 are used with the SVM.
- *RQ3*: What is the optimal MRI brain tumor analysis model using a hybrid machine learning approach?

Most papers (62.5%) discuss SVM's implementation, limitations, and advantages. Specifically, Senan et al. (2022) compared three different approaches and identified the combination of SVM and the hybrid model as the most promising.

## 5 Discussion

This section interprets the key findings and explores the implications of hybrid ML/DL models for brain tumour detection, structured across major themes such as technical efficacy, clinical relevance, interpretability, and implementation challenges.

### 5.1 Technical efficacy and diagnostic power

Hybrid models demonstrated superior performance compared to single-method approaches. Integrating traditional ML (e.g., SVM) with DL (e.g., CNNs like VGG-19 or YOLOv10n) enabled:

- Improved feature extraction and pattern recognition from complex MRI data.
- Higher resistance to false positives and class imbalance, enhancing diagnostic reliability.
- Better computational efficiency in real-time environments, especially with models like YOLOv10n.

These benefits are particularly impactful in distinguishing between glioma, meningioma, and pituitary tumours, as reported by Senan et al. (2022) and Rasool et al. (2022).

### 5.2 Clinical utility and real-world applications

Hybrid models showed promise in accelerating diagnosis, supporting radiologists, and reducing diagnostic errors. Their use is particularly viable in:

- Triage systems that prioritize high-risk cases.
- Mobile or rural diagnostic units due to their low computational requirements (e.g., YOLOv10n).
- Supplementary decision support tools for improving detection sensitivity in early tumour stages.

However, clinical implementation remains limited due to gaps in integration with hospital systems and workflow interoperability.

### 5.3 Role of explainable AI in adoption

Interpretability remains a significant barrier to clinical acceptance. Few reviewed studies applied Explainable AI (XAI) techniques such as:

- LIME (Local Interpretable Model-Agnostic Explanations)
- SHAP (SHapley Additive exPlanations)

Models that incorporated XAI (e.g., VGG-19 + SHAP) produced more transparent decision pathways, allowing radiologists to understand why a tumour was classified as malignant or benign (Park and Kim, 2024).

### 5.4 Challenges and limitations

Despite promising results, several challenges persist:

*Data Limitations*: Most studies used homogeneous datasets with limited variability, reducing generalizability.
*Lack of Standardization*: Inconsistent evaluation metrics, training-validation splits, and reporting practices hinder direct model comparison.
*Limited Real-Time Testing*: Most models were tested under controlled, retrospective conditions, with few deployed in prospective clinical settings.
*Ethical and Regulatory Concerns*: Few studies addressed data privacy, algorithmic bias, or compliance with medical device regulations.

### 5.5 Priority for the future of research

To enable broader adoption of hybrid models in clinical settings, future work should prioritize:

- Standardized benchmarks and open annotated datasets for fair comparison.
- Interdisciplinary collaboration between clinicians, radiologists, and AI researchers.
- Integration of XAI tools to improve transparency and trust.
- End-to-end deployment pipelines that include image acquisition, preprocessing, classification, and clinical feedback loops.

## 6 Conclusion

This review has highlighted hybrid machine learning models’ growing relevance and performance benefits in brain tumour detection from MRI images. By analysing various studies, it becomes evident that combining the strengths of conventional ML models like SVM with deep learning models such as VGG-19 and YOLOv10n significantly enhances classification accuracy, computational efficiency, and robustness. These hybrid systems outperform standalone models by leveraging CNNs’ feature extraction capabilities alongside the decision boundaries offered by traditional classifiers.

Among the evaluated models, the SVM + VGG-19 hybrid demonstrated superior diagnostic performance, while YOLOv10n offered real-time inference benefits for segmentation tasks. Nonetheless, the adoption of such models in clinical environments is limited by challenges including data scarcity, model overfitting on small or homogeneous datasets, and insufficient integration of explainable AI mechanisms for transparency and trustworthiness in decision-making.

This review provides valuable insights into the effectiveness of hybrid ML and DL models in MRI brain tumour detection, offering a structured evaluation of existing methodologies and future research directions. Nevertheless, clinical image analysis, particularly detecting structures within clinical images using computational methods, is a rapidly evolving and growing discipline. Image detection is central to identifying critical regions of interest for diagnosis and treatment planning. Despite significant advancements, challenges remain due to inherent anatomical variations. The emergence of deep neural networks has revolutionized the field, delivering cutting-edge results in medical image detection. However, these methods have limitations, including reliance on deterministic predictions, limited interpretability, and the need for large datasets. In the medical domain, where accuracy and reliability are vital, prediction errors can lead to serious consequences.

### 6.1 Clinical emphasis

Beyond performance metrics, the true value of hybrid machine learning models lies in their potential to enhance clinical workflows and support timely, accurate diagnosis of brain tumours. This review shows that these models can significantly reduce computational burden and improve diagnostic performance, but also emphasizes that clinical applicability requires more than just algorithmic success.

Future efforts must prioritize developing models that are accurate and generalizable across diverse populations, compatible with clinical systems, and transparent enough to gain the trust of clinicians. Collaborations with healthcare professionals during the model development process and pilot testing in real clinical environments will be essential to ensure usability, safety, and ethical compliance.

Adopting lightweight, explainable, and clinically validated hybrid models can ultimately contribute to earlier diagnosis, personalized treatment planning, and improved patient outcomes, particularly in under-resourced healthcare settings. As such, hybrid models are not just a technical advancement, but a potential catalyst for more equitable and efficient cancer care.

### 6.2 Future research directions

Future research should prioritize the development of standardized benchmark datasets, the integration of advanced explainable AI (XAI) frameworks such as LIME and SHAP, and the creation of end-to-end pipelines that are both accurate and resource-efficient while maintaining interpretability. In addition, exploring the prospects of hybrid models by incorporating transfer learning and ensemble voting strategies would be highly beneficial. These approaches can enhance model generalizability, robustness, and predictive performance, especially in scenarios with limited annotated medical data.

Moreover, interdisciplinary collaboration between medical professionals and data scientists remains crucial to ensure that developed models meet clinical standards, ethical guidelines, and real-world usability requirements. By focusing on these areas, future work can contribute to advancing intelligent, transparent, and clinically viable solutions for brain tumour detection.

## Data Availability

The original contributions presented in the study are included in the article/supplementary material, further inquiries can be directed to the corresponding author.
